# Macroscopic anomaly detection in paddy fields from UAV perspectives: a frequency-aware spatiotemporal mamba approach

**DOI:** 10.3389/fpls.2026.1895982

**Published:** 2026-08-18

**Authors:** Yi Liu, Hongzhi Cui, Junjie He, Jinping Xiao

**Affiliations:** 1 School of Artificial Intelligence, China University of Mining and Technology (Beijing), Beijing, China; 2 Beijing Aerospace Changfeng Science Technology Industry Group Co., Ltd, Beijing, China

**Keywords:** frequency-domain feature learning, future-frame prediction, normal-only learning, paddy-field monitoring, state-space modeling, unmanned aerial vehicle remote sensing, video anomaly detection

## Abstract

Unmanned aerial vehicles (UAVs) provide high-resolution and temporally continuous observations, offering substantial value for wide-area paddy-field inspection. Timely detection of macroscopic anomalies such as fire, intrusion, lodging, and disease is important for field management and risk assessment. However, supervised methods rely on predefined categories and large numbers of annotated abnormal samples, which are difficult to collect comprehensively in field environments. Meanwhile, UAV ego-motion, viewpoint changes, repetitive crop textures, and multi-scale appearance variations further increase the difficulty of anomaly detection, limiting the direct application of conventional image-based and fixed-camera video methods to moving UAV videos. To address these challenges, this paper proposes TFMAD, a normal-only futureframe prediction framework for frame-level macroscopic anomaly detection in UAV paddy-field videos. TFMAD adopts a U-shaped encoder–decoder architecture, learns regular spatiotemporal patterns only from normal videos, and identifies anomalies according to the discrepancy between the predicted and observed frames. The framework contains three core modules: the Frequency Spatiotemporal Correlation Module (FSCM) models time–frequency variation and spatial correlation; the Parallel Spatiotemporal Mamba (PSTM) module models temporal variation and local structures through bidirectional pixel-level and block-level scanning; and the DifferenceGuided Fusion (DGF) module regulates skip connections according to the discrepancy between appearance and spatiotemporal features, reducing the direct transfer of abnormal information to the decoder. To balance high-resolution feature extraction and computational efficiency, the encoder further adopts Token Statistics Self-Attention (TSSA) with linear complexity. In addition, this study constructs URVAD, a UAV paddy-field video dataset for frame-level anomaly detection, and conducts experiments on this dataset. The results show that TFMAD achieves 95.83% Micro-AUC and 96.80% Macro-AUC, obtaining the best overall performance among the compared methods under the current dataset and experimental setting.

## 1 Introduction

Rice production is constrained by biotic stresses and weather-related abiotic stresses, including diseases, insect pests, drought, temperature extremes, and flooding (Vo et al., 2021; Radha et al., 2023). The occurrence and severity of these stresses can vary spatially, resulting in localized patterns of crop damage or growth deterioration within rice-growing areas (Bellis et al., 2022; Amoghavarsha et al., 2022). Timely field observation is therefore important for identifying crop damage and operational risks before they spread or intensify. Unmanned aerial vehicles (UAVs) have become an important remote-sensing platform in precision agriculture because they can acquire high-resolution observations on demand, revisit fields repeatedly, and cover areas that are laborious or costly to inspect manually (Shahi et al., 2022). For example, UAV-based multispectral imagery has been combined with ground-based hyperspectral measurements to estimate plant nitrogen concentration in rice (Zheng et al., 2018). UAV hyperspectral imagery acquired at multiple growth stages has also been used to estimate rice yield (Wang et al., 2019). In addition, multispectral UAV observations acquired at different flight altitudes have been evaluated for rice growth monitoring and yield prediction (Luo et al., 2022). Together, these studies demonstrate that UAV sensing can characterize field-scale variation in crop growth, nutritional status, and production traits. These capabilities make UAVs suitable for paddy-field inspection, particularly when changes must be observed from a field-scale perspective rather than through isolated close-range samples.

Many agricultural computer-vision systems formulate monitoring as supervised image classification, object detection, or semantic segmentation. Representative applications include crop-type classification from multispectral imagery (Deng et al., 2024), semantic segmentation of large-scale aerial agricultural patterns (Chiu et al., 2020), and plant localization, counting, or planting-gap identification from UAV imagery (Chen et al., 2017; Cai et al., 2022; Waqar et al., 2024). Related methods have also been developed to extract lodged rice areas (Zhao et al., 2019), map weeds (Yu et al., 2022), and identify crop diseases such as wheat yellow rust and bacterial blight in wild rice (Pan et al., 2021, 2023). Such methods and datasets are valuable when the target categories are known and sufficiently annotated, but their task assumptions differ from open-ended field monitoring. They learn decision boundaries for predefined classes, and their performance depends on whether the training data adequately represent the relevant crop varieties, growth stages, environmental conditions, sensing platforms, and target appearances (Angarano et al., 2024).

This dependence is restrictive for comprehensive paddy-field monitoring. Fires, bird or vehicle intrusions, severe lodging, and other unusual events are difficult to collect exhaustively in advance (Liu et al., 2018; Shikhar and Sobti, 2024). Localized appearance anomalies such as yellowing and blight, as well as structural anomalies such as lodging, may also vary substantially in their visual manifestation, spatial extent, and severity (Mandal et al., 2023). In addition, a single image does not explicitly indicate whether a visible change is persistent, progressively developing, or merely caused by normal illumination variation or camera movement (Liu et al., 2018; Jin et al., 2022). These limitations motivate a formulation that can exploit temporal context without requiring abnormal samples from every possible category during training. The present work therefore does not position its dataset as a replacement for established agricultural image datasets; instead, it studies a different task in which normal UAV video is used to identify temporally unexpected observations at the frame level.

This task naturally aligns with normal-only video anomaly detection, in which a model learns regularities from normal training videos and assigns higher anomaly scores at test time to observations that depart from those regularities (Zhu et al., 2024b). Among the normality-modeling approaches most relevant to this work, reconstruction and future-frame prediction are two foundational paradigms. Reconstruction-based methods learn to reproduce normal inputs and use reconstruction discrepancies as anomaly cues (Hasan et al., 2016), whereas prediction-based methods estimate a future frame from preceding observations and compare the prediction with its observed counterpart (Liu et al., 2018). Future-frame prediction became a representative baseline because events inconsistent with learned normal temporal dynamics are expected to be more difficult to predict accurately. Subsequent methods introduced memory banks or prototype units to represent diverse normal patterns and constrain the features available for reconstruction or prediction, thereby reducing the risk of accurately reproducing abnormal content (Park et al., 2020; Lv et al., 2021). Hybrid designs have also combined motion reconstruction with frame prediction so that deviations in appearance and motion can jointly influence the anomaly score (Liu et al., 2021).

Despite this progress, many widely used video anomaly detection benchmarks — and the methods developed and evaluated on them — focus on footage captured by stationary ground surveillance cameras. Because these datasets are typically recorded from fixed viewpoints, the background geometry remains largely stable within each scene, and annotated anomalies are commonly associated with unusual foreground objects or actions. These assumptions do not transfer directly to videos acquired by a moving UAV. Camera translation, altitude changes, and viewpoint variation can induce global image motion, scale changes, and viewpoint-dependent appearance variation (Jiao et al., 2023; Jin et al., 2022). Consequently, normal frame-to-frame differences in UAV videos may arise from platform motion rather than anomalous events. Meanwhile, the anomalies of interest exhibit heterogeneous temporal characteristics. Vehicles and birds may produce salient object motion, whereas fire and smoke can introduce conspicuous and continuously evolving appearance changes. By contrast, lodging, yellowing, and blight may emerge through slower changes in canopy structure, color, or texture (Mandal et al., 2023; Pan et al., 2023). A model that reacts too strongly to temporal variation may respond to normal platform-induced changes, whereas a model that is overly invariant to slowly evolving patterns may reduce its sensitivity to gradual agricultural anomalies.

Aerial video anomaly detection has begun to address some of the difficulties introduced by moving platforms. Drone-Anomaly and its associated transformer-based prediction model demonstrated that normality learning and future-frame prediction can be extended to aerial scenes (Jin et al., 2022). UITADrone subsequently provided a drone-based benchmark for traffic anomaly detection, and a transformerbased spatiotemporal prediction method was developed to model normal traffic patterns in aerial videos (Tran et al., 2023, 2024). More recently, HSTforU embedded hierarchical spatiotemporal transformers into a U-shaped prediction network and evaluated the resulting model across aerial and ground-based video anomaly detection benchmarks (Le et al., 2025). In parallel with these transformer-based developments, selective state-space models have emerged as an efficient alternative for long-sequence modeling. Mamba introduced input-dependent selective state-space operations with linear scaling in sequence length (Gu and Dao, 2024). For visual representation learning, Vision Mamba employed bidirectional state-space modeling, while VMamba introduced multi-directional two-dimensional selective scanning to aggregate spatial context from different traversal directions (Zhu et al., 2024a; Liu et al., 2024). VideoMamba further extended state-space modeling to video understanding by using linear-complexity operations to capture long-range spatiotemporal dependencies (Li et al., 2024a). Mamba-based architectures have subsequently been introduced into video anomaly detection. STNMamba learns spatial and temporal normality through dedicated state-space encoders, whereas VADMamba combines Mamba-based feature modeling with future-frame prediction and optical-flow reconstruction (Li et al., 2024b; Lyu et al., 2025). These studies provide important evidence and comparison points for aerial video analysis and Mamba-based normality modeling. However, limited attention has been given to normal-only anomaly detection in moving UAV paddy-field videos. In this setting, UAV ego-motion continuously changes the observed background, while anomalous objects or regions may enter and leave the field of view during flight. The main challenge is therefore to distinguish normal scene variations caused by platform motion from localized observations that deviate from regular paddy-field appearance or motion patterns. This motivates task-specific spatiotemporal modeling for frame-level anomaly detection in moving UAV paddy-field videos.

To this end, we propose TFMAD, a U-shaped future-frame prediction network for normal-only UAV paddy-field video anomaly detection. The encoder adopts Token Statistics Self-Attention (TSSA) (Wu et al., 2024), an existing linear-time attention operator, to process high-resolution features with controlled computational cost. At each skip level, the Frequency Spatiotemporal Correlation Module (FSCM) weights temporal-frequency components and models spatiotemporal autocorrelation, while the Parallel Spatiotemporal Mamba (PSTM) module uses complementary forward and reverse pixel-level and blocklevel scanning paths to capture directional dependencies and local structures. The Difference-Guided Fusion (DGF) module compares the latest-frame encoder representation with the refined spatiotemporal representation and uses the resulting discrepancy to regulate skip-feature transfer. The model is trained only with normal sequences, and frame-level anomaly scores are derived from the discrepancy between the predicted and observed future frames.

The main contributions are summarized as follows:

1. We formulate UAV paddy-field monitoring as a normal-only, frame-level video anomaly detection problem, enabling the evaluation of heterogeneous and separately represented macroscopic anomaly scenarios without requiring annotated abnormal samples during training.
2. We develop TFMAD, which combines an adopted linear-time TSSA encoder with parallel FSCM and PSTM skip-refinement branches and DGF-based skip regulation in the decoder. The design jointly considers temporal-frequency variation, multi-directional spatiotemporal dependencies, and controlled skip-feature transmission; TSSA itself is used as an existing efficient attention operator rather than claimed as an original contribution.
3. We curate and use the URVAD dataset as a task-specific evaluation set containing normal UAV paddy-field training videos and six separately represented anomaly categories with frame-level test annotations. These categories cover moving-object intrusions, fire, canopy-structure anomalies, and crop-appearance anomalies. Under this evaluation setting, TFMAD achieves 95.83% Micro-AUC and 96.80% Macro-AUC, while the ablation results quantify the contributions and trade-offs of its principal components.

## 2 Materials and methods

### 2.1 Data collection and preprocessing

To evaluate the effectiveness of normal-only learning for anomaly detection in UAV paddy-field videos, we constructed a task-specific UAV video anomaly dataset named URVAD. Multilingual keywords, such as rice paddy aerial view, were used to extensively retrieve and collect raw aerial footage from mainstream video-sharing platforms and other sources. The collected materials cover multi-scale top-down views acquired at different flight altitudes. To improve the validity of the dataset and reduce interference from irrelevant visual factors, the collected footage was screened according to the following criteria. We excluded: (1) videos that did not depict paddy-field scenes or in which the rice canopy could not be clearly identified; (2) manually edited videos containing transitions, overlays, or other post-production effects; and (3) segments with severe camera shake that prevented reliable representation of spatiotemporal continuity.

After screening and temporal segmentation, URVAD contains 13 video clips, including seven normal training clips and six test clips. Before being resized to 256 × 256 pixels for model input, all retained clips have a native spatial resolution of 640 × 640 pixels and a frame rate of 30 fps. All clips were acquired during daytime under natural daylight and clear-weather conditions. The frame counts and durations of the individual clips are reported in **Table 1**, while the numbers of normal and anomalous frames and the corresponding anomaly-frame ratios are summarized in **Table 2**.

**Table 1:** Video-level composition of the URVAD dataset.

Split	Clip ID	Category	Frames	Duration (s)
Training	Train-01	Normal	995	33.17
Training	Train-02	Normal	311	10.37
Training	Train-03	Normal	658	21.93
Training	Train-04	Normal	810	27.00
Training	Train-05	Normal	227	7.57
Training	Train-06	Normal	622	20.73
Training	Train-07	Normal	1007	33.57
Training total			4630	154.33
Testing	Test-01	Vehicle	361	12.03
Testing	Test-02	Fire	241	8.03
Testing	Test-03	Lodging	192	6.40
Testing	Test-04	Bird	289	9.63
Testing	Test-05	Yellowing	241	8.03
Testing	Test-06	Blight	241	8.03
Testing total			1565	52.17

**Table 2:** Detailed statistics of the URVAD dataset, including normal and anomalous frame counts, total evaluated frames, and anomaly-frame ratios.

Split	Video/category	Normal	Anomalous	Total	Anomaly ratio
Training	Normal paddy background	4630	0	4630	0.00%
Testing	Vehicle	12	349	361	96.68%
	Fire	82	159	241	65.98%
	Lodging	35	157	192	81.77%
	Bird	63	226	289	78.20%
	Yellowing	75	166	241	68.88%
	Blight	30	211	241	87.55%
Testing total	Six test categories	297	1268	1565	81.02%

Following the standard normal-only video anomaly detection setting, URVAD is divided into a training set containing only normal scenes and a test set with frame-level anomaly annotations.

**Figure 1:**
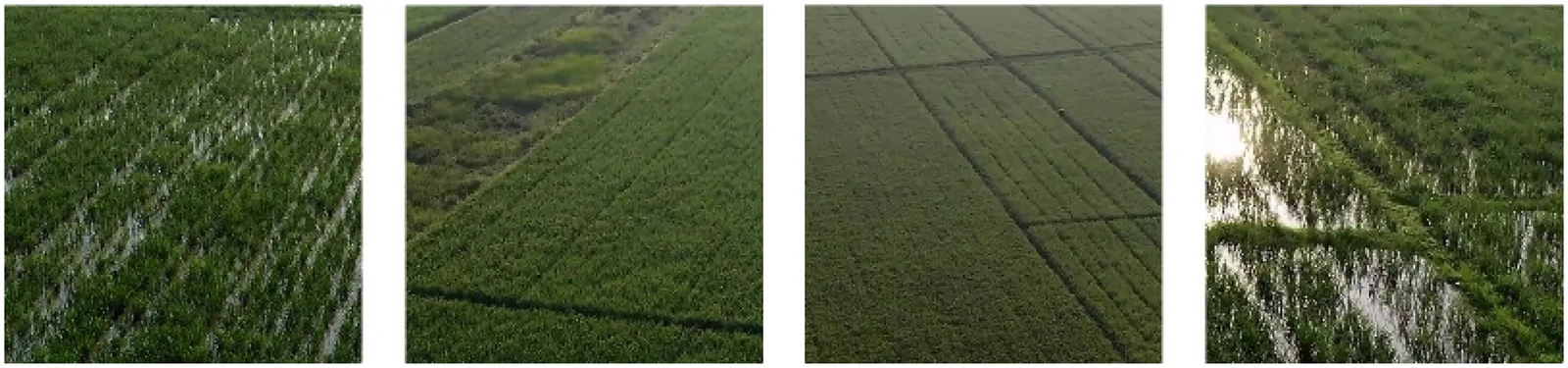
Training set examples. Displays normal wide-area paddy field perspectives captured at various flight altitudes under different camera viewpoints.

Training Set: The training set contains only normal paddy-field scenes. Specifically, seven nonoverlapping clips were extracted from different temporal portions of one long normal UAV paddy-field video. As shown in **Figure 1**, these clips contain only normal paddy-field backgrounds while exhibiting variations in UAV viewpoint, flight altitude, and crop-canopy appearance.

**Figure 2:**
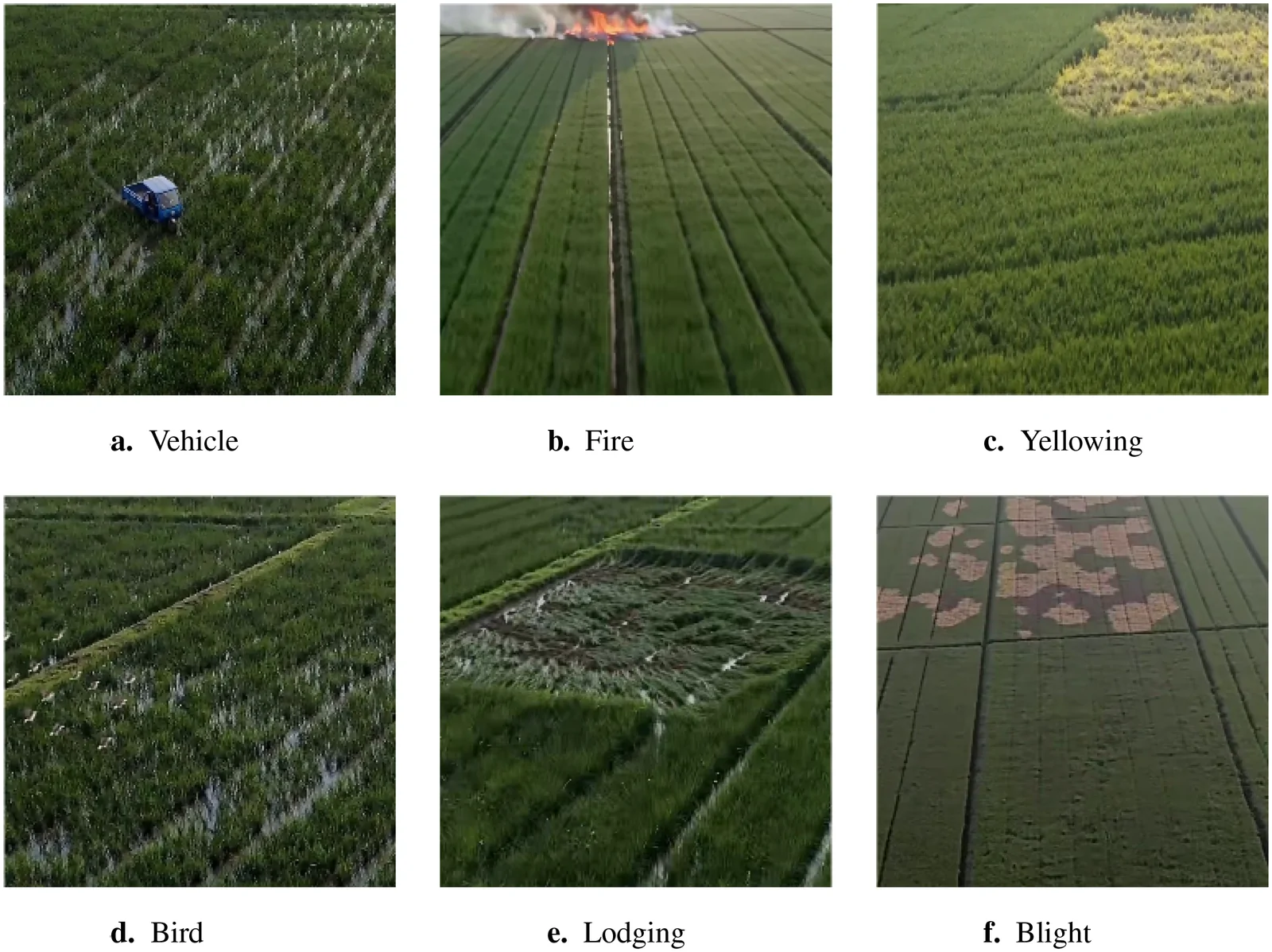
Testing set anomaly examples. **(a)** Vehicle. **(b)** Fire. **(c)** Yellowing. **(d)** Bird. **(e)** Lodging. **(f)** Blight.

Testing Set: The test set consists of six separately collected video clips, each corresponding to one macroscopic anomaly category. As shown in **Figure 2**, the six categories are Vehicle, Fire, Lodging, Bird, Yellowing, and Blight.

Because the model is trained exclusively on normal scenes, the training clips do not require frame-level anomaly annotations. For the test set, frame-level annotations were manually prepared for temporal anomaly detection evaluation. A frame was labeled as anomalous (1) if any of the aforementioned paddy-field anomalies appeared; otherwise, it was labeled as normal (0) if it contained only a normal paddy-field background.

### 2.2 Method

#### 2.2.1 TFMAD network architecture

To address the high cost and low efficiency of manually inspecting large-scale rice paddies, this study introduces an unsupervised video anomaly detection framework for UAV-based rice paddy monitoring. The framework requires only normal video sequences for training; by learning the spatiotemporal regularities of normal scenes, it identifies frames that deviate from the learned patterns as anomalies during inference, enabling the detection of various macroscopic events (e.g., fire, crop lodging, foreign-object intrusion) without large-scale annotation of anomalous samples.

**Figure 3:**
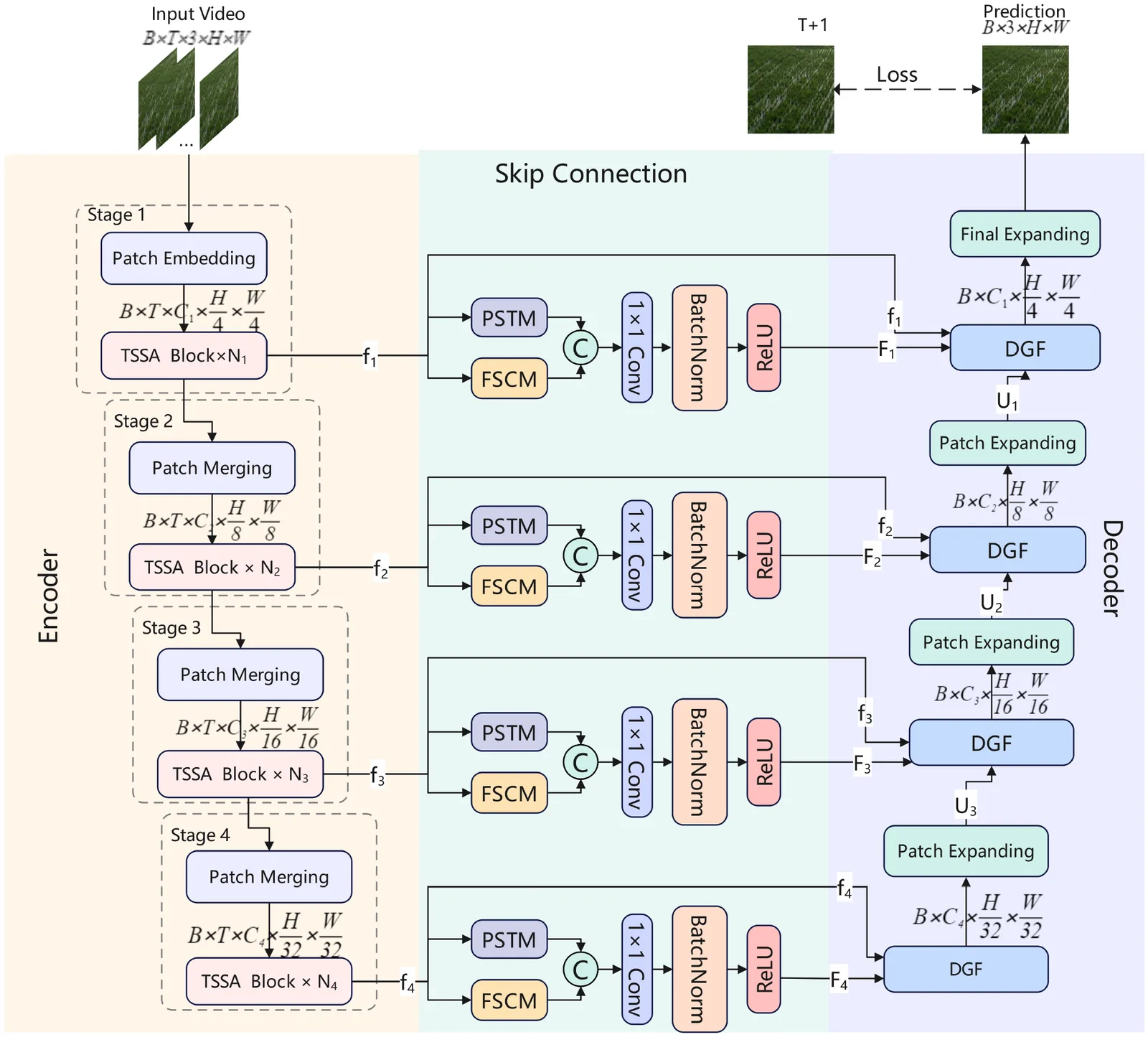
The overall spatiotemporal prediction network architecture of TFMAD. The model adopts an encoder-decoder structure, utilizing TSSA blocks for feature extraction, FSCM and PSTM for dualbranch feature refinement at the skip connections, and DGF modules in the decoder to regulate the direct transmission of skip features.

Based on this framework, we construct a future-frame prediction network termed TFMAD, as illustrated in **Figure 3**. Given *T* consecutive RGB frames, the network predicts the frame at time *T* + 1, and the discrepancy between the predicted and observed frames is used to compute the anomaly score. TFMAD consists of a multi-stage encoder, skip-connection refinement branches, and a progressively upsampling decoder. The encoder hierarchically extracts multi-scale spatiotemporal features, with TSSA blocks incorporated at each stage for feature enhancement.

Let *f_i_* denote the output of the *i*-th encoder stage. As shown in **Figure 3**, *f_i_* is transmitted through two paths. One path provides the latest-frame encoder feature to the corresponding DGF module, while the other feeds the full spatiotemporal feature into the parallel FSCM and PSTM branches for frequencydomain correlation modeling and spatiotemporal scanning, respectively. Because the branch outputs retain the temporal dimension whereas DGF operates on spatial skip features, the outputs of the FSCM and PSTM branches are first reshaped by merging the temporal and channel dimensions into a single channel dimension. The two reshaped outputs are then concatenated along the channel dimension and projected by a convolution that restores the channel number to match the latest-frame encoder feature, producing the refined feature *F_i_*. The resulting *F_i_* is aligned with the encoder feature in channel and spatial dimensions, and both are subsequently passed to the corresponding DGF module together with the upsampled decoder feature.

The decoder progressively upsamples the feature representations and integrates them with the corresponding encoder and refined features through the DGF modules. After the final decoding stage, the feature map is restored to the original spatial resolution to generate the predicted frame. The detailed structures of TSSA, FSCM, PSTM, and DGF are introduced in the following subsections.

#### 2.2.2 TSSA

The computational complexity of standard self-attention grows quadratically with the number of tokens, imposing a substantial burden when processing high-resolution feature maps from UAV aerial videos. To address this, we adopt the Token Statistics Self-Attention (TSSA) module proposed by Wu et al. (2024) and integrate it into every stage of the TFMAD encoder as a drop-in replacement for standard self-attention. The original structure and mathematical formulation of TSSA are kept exactly as in (Wu et al., 2024); hence, TSSA itself is not an original methodological contribution of this work, but rather serves as an efficient attention mechanism within the proposed TFMAD framework.

TSSA originates from algorithm unrolling applied to the variational form of the Maximal Coding Rate Reduction (MCR^2^) objective. Unlike standard self-attention, which computes an attention matrix via pairwise token similarities, TSSA models token representations through the second-moment statistics of projected features.

Let $Z=\left[z_{1},z_{2},\ldots ,z_{N}\right]\in {\mathbb{R}}^{d\times N}$ denote the input token matrix, where *N* is the number of tokens, *d* is the token feature dimension, and $z_{j}\in {\mathbb{R}}^{d}$ is the *j*-th token. Let *K*_att_ be the number of attention heads, *p* (*p* ≤ *d*) the projection dimension per head, and $U_{k}\in {\mathbb{R}}^{d\times p}$ the learnable projection matrix of the *k*-th head. *η* > 0 is a learnable temperature parameter. As shown in Equation 1, The subspace membership probability vector of token *z_j_* is 220 defined as

(1) $$\nu \left(z_{j}\right)=\text{softmax }\left(\frac{1}{2\eta }{\left[\|U_{1}^{⊤}z_{j}{\|}_{2}^{2},\ldots ,\|U_{K}^{⊤}z_{j}{\|}_{2}^{2}\right]}^{⊤}\right),$$

where $\nu \left(z_{j}\right)\in {\mathbb{R}}^{K_{\text{att}}}$ contains the membership probabilities of the *j*-th token with respect to the *K*_att_ projected subspaces. The membership vectors of all tokens form the membership matrix $\prod \in {\mathbb{R}}^{N\times K_{\text{att}}}$, whose *k*-th column is denoted by $\pi_{k}=\prod_{:,k}\in {\mathbb{R}}^{N}$.

As shown in Equation 2, Based on this membership matrix, the TSSA operator is given by

(2) $$\text{TSSA }(Z)=-\sum_{k=1}^{K_{\text{att}}}U_{k}D(Z,\pi_{k}|U_{k})U_{k}^{⊤}Z\text{Diag }(\pi_{k}),$$

where $D(Z,\pi_{k}|U_{k})\in {\mathbb{R}}^{p\times p}$ is a diagonal weighting matrix constructed from the second-moment statistics of the tokens projected onto the *k*-th subspace, and $\text{Diag }(\pi_{k})\in {\mathbb{R}}^{N\times N}$ is the diagonal matrix formed from *π_k_*. The diagonal entries of $D(Z,\pi_{k}|U_{k})$ decrease as the energy along the corresponding projection directions increases. Consequently, the operator performs a data-dependent approximate low rank projection that preserves feature directions with stronger second-moment responses while suppressing weaker ones.

Because TSSA avoids explicit pairwise token interactions, its computational cost grows linearly with the number of tokens *N* (for fixed feature dimensions and number of heads), in contrast to the quadratic growth of standard self-attention. This property makes TSSA particularly suitable for high-resolution UAV feature maps. In TFMAD, we embed the TSSA module into each encoder stage to enhance feature representation with controlled computational overhead.

#### 2.2.3 FSCM

**Figure 4:**
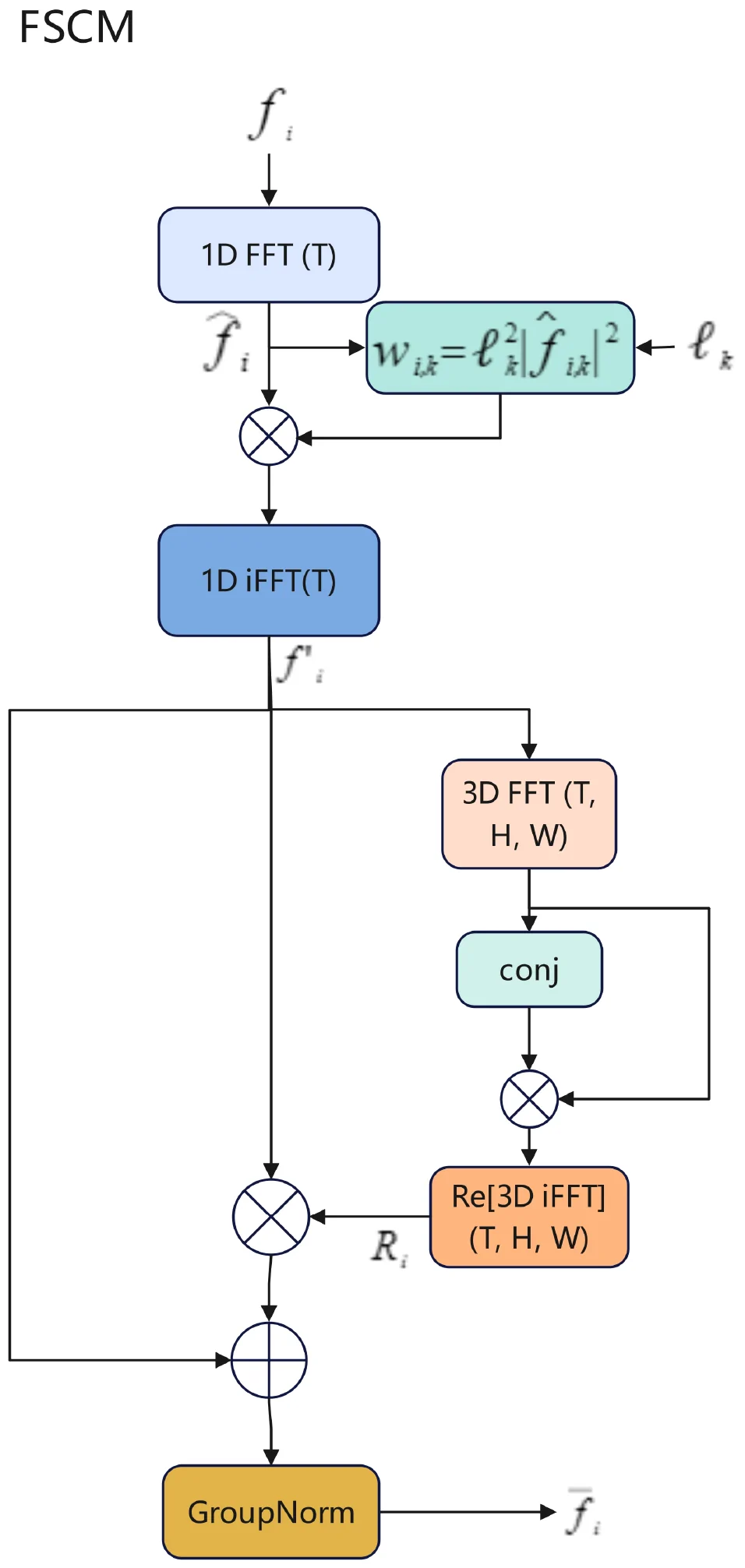
Detailed structure of the Frequency Spatiotemporal Correlation Module (FSCM).

In UAV-based rice paddy inspection, the global background displacement caused by UAV ego-motion is spatially coupled with the local motion of anomalous regions, making it difficult to distinguish the two motion sources directly in the spatial domain. Frequency-domain representations offer an alternative perspective, as different motion patterns typically exhibit different spectral characteristics. We construct a Frequency Spatiotemporal Correlation Module (FSCM), whose structure is illustrated in **Figure 4**.

Let $f_{i}\in {\mathbb{R}}^{B\times T\times C_{i}\times H_{i}\times W_{i}}$ be the feature produced by the *i*-th encoder stage, where *B*, *T*, *C_i_*, *H_i_*, and *W_i_* denote the batch size, temporal length, number of channels, height, and width, respectively. FSCM first applies a one-dimensional Fourier transform along the temporal dimension, yielding the complexvalued frequency representation ${\hat{f}}_{i}={ℱ}_{T}\left(f_{i}\right)$. The *k*-th temporal frequency component is denoted by ${\hat{f}}_{i,k}\in {\mathbb{C}}^{B\times C_{i}\times H_{i}\times W_{i}}$, with k=0,1,…,T−1.

As shown in Equation 3, A normalized temporal frequency coordinate *ℓ_k_* is defined as

(3) $$\ell_{k}=\{\begin{array}{ll} k/T, & 0\leq k\leq \left[T/2\right],\\ \left(k-T\right)/T, & \left[T/2\right]<k\leq T-1. \end{array}$$

As shown in Equation 4, Using the magnitude $\left|{\hat{f}}_{i,k}\right|$ and the frequency coordinate, a frequency-adaptive weight is computed for each component:

(4) $$w_{i,k}=\ell_{k}^{2}{\left|{\hat{f}}_{i,k}\right|}^{2},$$

where the square and multiplication are performed element-wise, and $\ell_{k}^{2}$ is broadcast over the batch, channel, and spatial dimensions. The weight *w_i_*,*_k_* emphasizes components with both larger normalized frequency and stronger spectral energy.

As shown in Equations 5, 6, Each frequency component is then modulated by its corresponding weight:

(5) $${\hat{f}}_{i,k}^{\prime }={\hat{f}}_{i,k}⊙w_{i,k},$$

where ⊙ denotes element-wise multiplication. After reassembling the weighted components into the full spectral tensor ${\hat{f}}_{i}^{\prime }$, an inverse Fourier transform along the temporal axis recovers a frequency-weighted feature in the time domain:

(6) $$f_{i}^{\prime }=\text{Re}\left[{ℱ}_{T}^{-1}\left({\hat{f}}_{i}^{\prime }\right)\right],$$

with $f_{i}^{\prime }\in {\mathbb{R}}^{B\times T\times C_{i}\times H_{i}\times W_{i}}\;$ and Re(·) taking the real part.

Subsequently, FSCM models correlations directly over the complete spatiotemporal volume. As shown in Equation 7, For each batch sample and each feature channel, a three-dimensional Fourier transform is applied along the (*T,H_i_,W_i_*) dimensions:

(7) $$G_{i}={ℱ}_{THW}\left(f_{i}^{\prime }\right),$$

giving the complex-valued frequency tensor $G_{i}\in {\mathbb{C}}^{B\times T\times C_{i}\times H_{i}\times W_{i}}$. As shown in Equation 8, Its power spectral density is obtained via

(8) $$P_{i}=G_{i}⊙G_{i}^{*},$$

where (·)^∗^ denotes complex conjugation. As shown in Equation 9, By the Wiener–Khinchin theorem (Wiener, 1930), the spatiotemporal autocorrelation tensor is computed through an inverse three-dimensional Fourier transform of *P_i_*:

(9) $$R_{i}=\text{Re }\left[{ℱ}_{THW}^{-1}\left(P_{i}\right)\right],$$

with $R_{i}\in {\mathbb{R}}^{B\times T\times C_{i}\times H_{i}\times W_{i}}$ and the transform including its standard normalization factor.

Finally, As shown in Equation 10, the autocorrelation tensor enhances the frequency-weighted feature via element-wise modulation and a residual connection, after which Group Normalization stabilizes the feature distribution:

(10) $${\bar{f}}_{i}=\text{GroupNorm }\left(f_{i}^{\prime }+R_{i}⊙f_{i}^{\prime }\right),$$

where ${\bar{f}}_{i}\in {\mathbb{R}}^{B\times T\times C_{i}\times H_{i}\times W_{i}}$ is the output of FSCM. In practice, Group Normalization is applied independently to the feature map at each temporal position.

#### 2.2.4 PSTM

**Figure 5:**
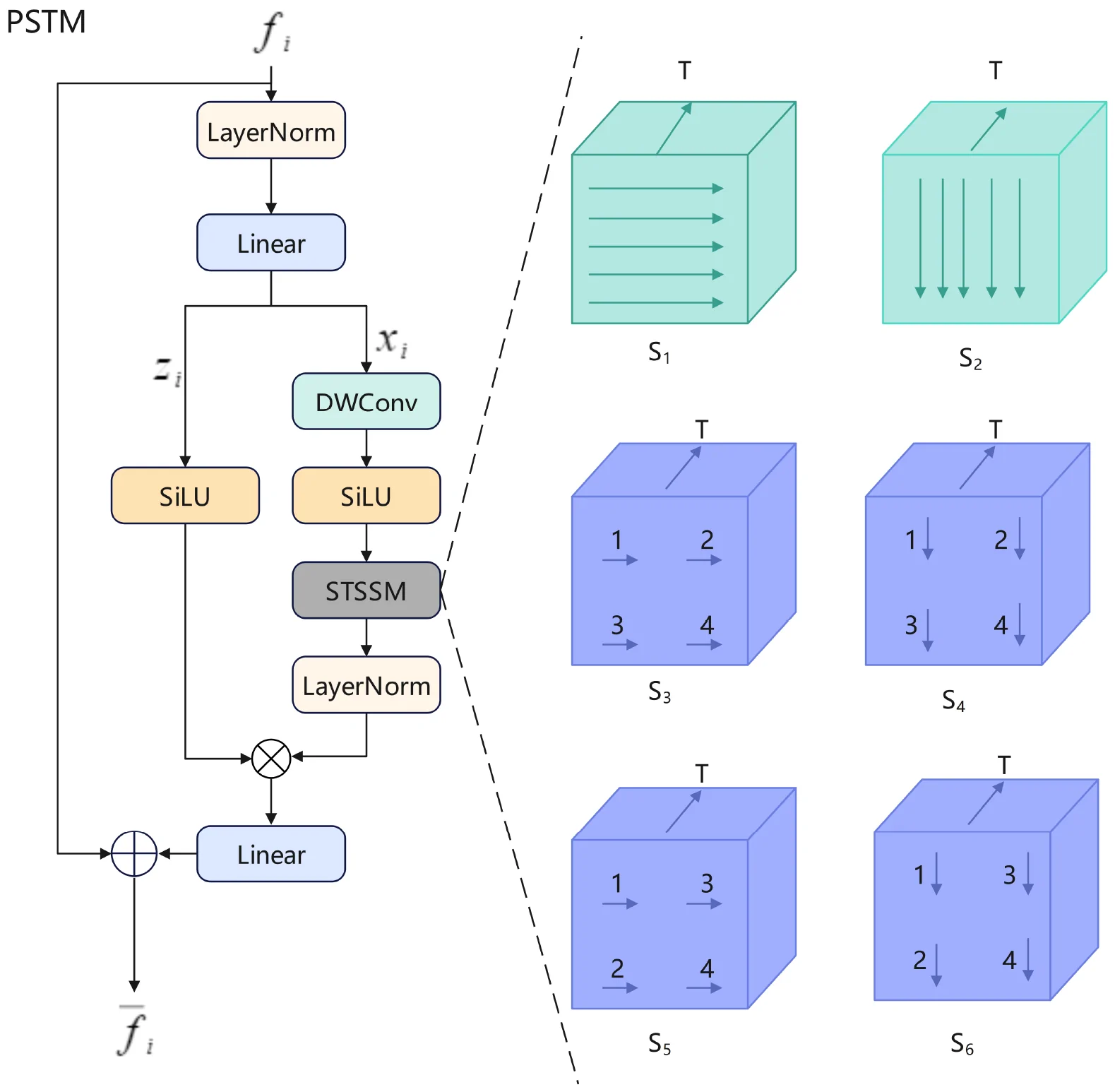
Detailed structure of the parallel spatiotemporal mamba (PSTM) module, illustrating the multi-path complementary scanning strategy.

Although FSCM efficiently captures global spatiotemporal dependencies in the frequency domain, it provides limited explicit modeling of directional temporal evolution and local spatial structures. To complement FSCM, we design a Parallel Spatiotemporal Mamba (PSTM) module that models encoder features through multiple complementary scanning paths, as illustrated in **Figure 5**.

As shown in Equation 11, Let $f_{i}\in {\mathbb{R}}^{B\times T\times C_{i}\times H_{i}\times W_{i}}$ denote the feature produced by the *i*-th encoder stage, where *B*, *T*, *C_i_*, *H_i_*, and *W_i_* denote the batch size, temporal length, channel number, height, and width, respectively. The input is first normalized and linearly projected, and the projected feature is equally divided along the channel dimension into a main-branch feature *x_i_* and a gating feature *z_i_*:

(11) $$\left[x_{i},z_{i}\right]=\text{Split}\left(\text{Linear}\left(\text{LayerNorm}(f_{i})\right)\right),$$

where $x_{i},z_{i}\in {\mathbb{R}}^{B\times T\times D_{i}\times H_{i}\times W_{i}}$, and *D_i_* denotes the internal channel dimension of PSTM. The two Linear blocks shown in **Figure 5** are separate learnable projections, although the same notation is used for simplicity.

As shown in Equation 12, In the main branch, a two-dimensional depthwise convolution is independently applied to each temporal frame to introduce local spatial information. The resulting feature is activated by SiLU and then fed into the Spatiotemporal State Space Scanning Module (STSSM). Meanwhile, the gating branch generates a modulation feature through another SiLU activation:

(12) $$u_{i}=\text{SiLU }\left(\text{DWConv }(x_{i})\right),\;g_{i}=\text{SiLU }(z_{i}),$$

where $u_{i},g_{i}\in {\mathbb{R}}^{B\times T\times D_{i}\times H_{i}\times W_{i}}$. The depthwise convolution operates only over the spatial dimensions (*H_i_,W_i_*) and does not mix information along the temporal dimension.

Since the selective state space operator processes one-dimensional sequences, STSSM converts *u_i_* into several sequences using the six scanning modes *S*_1_–*S*_6_ shown in **Figure 5**. As shown in Equation 13, Let

(13) $$s_{i,m}=S_{m}\left(u_{i}\right)\in {\mathbb{R}}^{B\times L_{i}\times D_{i}},\;L_{i}=TH_{i}W_{i},$$

where $S_{m}\left(\cdot \right)$ denotes the *m*-th scanning operation and m∈{1,…,6}. Specifically, *S*_1_ and *S*_2_ traverse each frame in row-major and column-major spatial orders, respectively, with the temporal axis as the outermost scanning level, and the resulting per-frame sequences are concatenated along the temporal axis. For *S*_3_–*S*_6_, the feature map of each frame is first divided into non-overlapping blocks of size *P*×*P*, where the block size *P* satisfies *P*|*H_i_* and *P*|*W_i_*; row-major and column-major block traversal are combined with row-major and column-major intra-block traversal to form four complementary block-level sequences, with the temporal axis nested between the block-level and intra-block-level traversal so that, for each spatial block, all *T* frames are scanned before advancing to the next block. In this way, *S*_1_–*S*_2_ capture pixel-wise temporal continuity across the entire frame, while *S*_3_– capture temporal continuity within local spatial blocks.

As shown in Equation 14, Each base sequence is paired with its reversed sequence:

(14) $$s_{i,m}^{\text{rev}}=\text{Reverse }(s_{i,m}),$$

where Reverse(·) reverses the sequence along its length dimension. Therefore, the complete STSSM contains *K* = 6 × 2 = 12 scanning paths. Only the six base scanning modes are displayed in **Figure 5**, while their reverse counterparts are omitted for clarity.

Each sequence is processed by a selective state space operator following the standard Mamba formulation (Gu and Dao, 2024). The detailed state-space equations are therefore not repeated here. In the full twelve-path configuration, different paths use independent SSM parameters.

As shown in Equation 15, For the reverse path, the input sequence is first reversed, processed by an independent selective state space operator, and the output is reversed again to restore alignment with the forward path. We denote this composite operation as

(15) $${\tilde{ℳ}}_{m}^{\text{rev}}\left(s_{i,m}\right)\;≜\text{ Reverse }\left({ℳ}_{m}^{\text{rev}}\left(\text{Reverse }\left(s_{i,m}\right)\right)\right),$$

where ${ℳ}_{m}^{\text{rev}}\left(\cdot \right)$ is the selective state space operator applied to the reversed input, and the outer Reverse(·) restores the output to the original sequence order so that it is element-wise aligned with the forward path output. This realignment step is necessary because the reverse path otherwise produces an output sequence whose order is inverted relative to the forward path, which would prevent a direct position-wise summation.

As shown in Equation 16, The outputs of all forward and reverse paths are then aggregated by element-wise summation:

(16) $$q_{i}=\sum_{m=1}^{6}\left[{ℳ}_{m}^{\text{fwd}}\left(s_{i,m}\right)+{\tilde{ℳ}}_{m}^{\text{rev}}\left(s_{i,m}\right)\right],$$

where ${ℳ}_{m}^{\text{fwd}}\left(\cdot \right)$ denotes the selective state space operator for the forward path, ${\tilde{ℳ}}_{m}^{\text{rev}}\left(\cdot \right)$ is defined in Eq. (15), and $q_{i}\in {\mathbb{R}}^{B\times L_{i}\times D_{i}}$. The aggregated sequence is subsequently reshaped to the original spatiotemporal dimensions to obtain the STSSM output $v_{i}\in {\mathbb{R}}^{B\times T\times D_{i}\times H_{i}\times W_{i}}$.

Finally,As shown in Equation 17, the STSSM output is normalized and modulated by the gating feature. A linear projection restores the channel dimension from *D_i_* to *C_i_*, followed by a residual connection with the original input:

(17) $${\bar{f}}_{i}=f_{i}+\text{Linear }\left(\text{LayerNorm }(v_{i})⊙g_{i}\right),$$

where ⊙ denotes element-wise multiplication and ${\bar{f}}_{i}\in {\mathbb{R}}^{B\times T\times D_{i}\times H_{i}\times W_{i}}$ is the output of PSTM. In

**Figure 5**, the multiplication and addition nodes denote element-wise gating and residual addition, respectively.

#### 2.2.5 DGF

**Figure 6:**
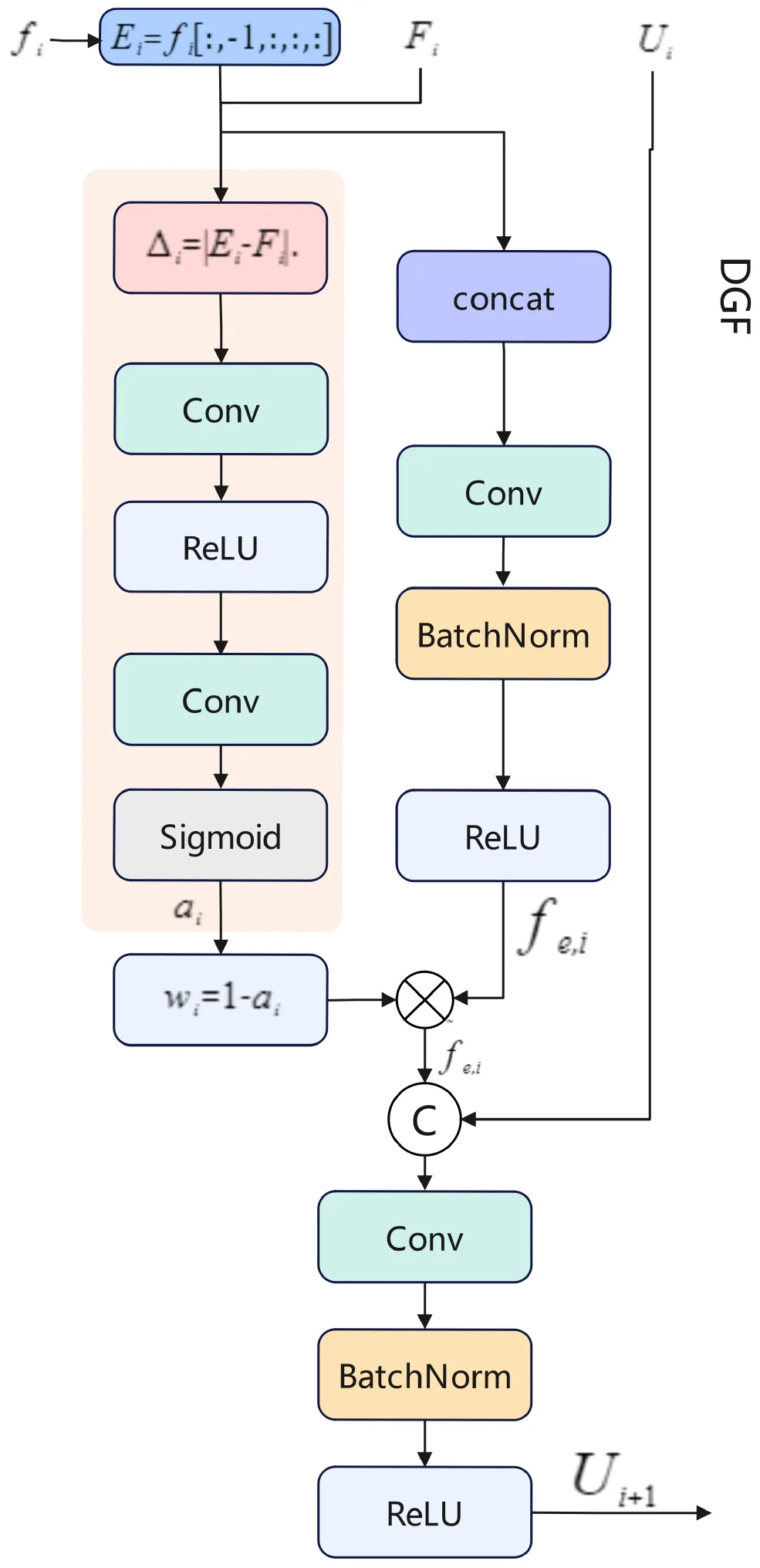
Detailed structure of the difference-guided fusion (DGF) module.

In future-frame prediction-based anomaly detection, conventional skip connections directly transfer encoder appearance features to the decoder. Although this operation preserves spatial details, it may also provide excessive appearance information for regions that deviate from the learned normal patterns, thereby reducing the prediction-error contrast between normal and anomalous regions. To regulate the transmission of skip features, we design a Difference-Guided Fusion (DGF) module, as illustrated in **Figure 6**. DGF uses the discrepancy between the latest encoder appearance feature and the refined spatiotemporal feature to generate a complementary gating map, which adaptively modulates the skip feature before decoder fusion.

For the *i*-th decoder stage, let $f_{i}\in {\mathbb{R}}^{B\times T\times C_{i}\times H_{i}\times W_{i}}$ denote the encoder feature, where *B*, *T*, *C_i_*, *H_i_*, and *W_i_* denote the batch size, temporal length, channel number, height, and width, respectively. DGF first selects the feature of the latest observed frame from *f_i_* as the appearance skip feature *E_i_*. The refined skip feature produced by the FSCM and PSTM branches is denoted as *F_i_*. Both *E_i_* and *F_i_* have size *B* × *C_i_* × *H_i_* × *W_i_* and are therefore aligned in channel and spatial dimensions. The upsampled decoder feature at the same spatial scale is denoted as *U_i_*.

DGF first concatenates *E_i_* and *F_i_* along the channel dimension and passes the concatenated feature through a convolution, Batch Normalization, and ReLU activation to obtain a fused skip feature. It then computes the element-wise absolute discrepancy between *E_i_* and *F_i_*. This discrepancy map is passed through two convolutional layers with ReLU and Sigmoid activations to generate a gate in the range [0,1]. The complementary gate is broadcast to match the fused skip feature when necessary and is multiplied with it before decoder fusion. The gated skip feature is finally concatenated with *U_i_* and fused by another convolution, Batch Normalization, and ReLU activation. At the deepest decoder stage, where no upsampled decoder feature is available, the fusion with *U_i_* is omitted.

Through this discrepancy-guided gating mechanism, DGF is designed to preserve appearance details from regions with consistent encoder and refined representations while reducing the direct transmission of features from high-discrepancy regions. This design encourages the decoder to use the learned temporal representation when predicting uncertain regions and is consistent with the reduced FPR observed in the ablation results.

#### 2.2.6 Loss function

This study employs a weighted combination of three loss functions for self-supervised future-frame prediction on normal training videos. The losses constrain the differences between the predicted frame *Y*_b_ and the target frame *Y* from three levels: pixel intensity, edge structure, and structural similarity.

Intensity Loss ${ℒ}_{int}$. As shown in Equation 18, The pixel-level *l*_2_ distance is adopted to penalize the numerical deviation between the predicted frame and the target frame:

(18) $${ℒ}_{int}\left(\hat{Y},Y\right)={\left\|\hat{Y}-Y\right\|}_{2}^{2}$$

Gradient Loss ${ℒ}_{grad}$. As shown in Equation 19, This loss constrains the edge structure of the predicted frame in the spatial gradient domain. It calculates the first-order differences of the predicted frame and the target frame in the horizontal and vertical directions, respectively, and penalizes the deviation between their gradient magnitudes:

(19) $${ℒ}_{grad}\left(\hat{Y},Y\right)=\sum_{i,j}{\left\|{\hat{Y}}_{i,j}-{\hat{Y}}_{i-1,j}\left|-\right|Y_{i,j}-Y_{i-1,j}\right\|}_{1}+{\left\|{\hat{Y}}_{i,j}-{\hat{Y}}_{i,j-1}\left|-\right|Y_{i,j}-Y_{i,j-1}\right\|}_{1}$$

where *i* and *j* indicate the spatial index of the frame. Compared with the pixel-level intensity term, ${ℒ}_{grad}$ emphasizes differences in local edge structure and paddy texture boundaries.

Structural Similarity Loss ${ℒ}_{ssim}$. As shown in Equation 20, Based on the SSIM metric, this loss measures the structural similarity between the predicted frame and the target frame in terms of luminance, contrast, and structure. This study adopts a dual-scale calculation method, calculating the SSIM at both the original resolution and a 2× downsampled resolution, and then taking the average to account for structural consistency across different scales:

(20) $${ℒ}_{ssim}\left(\hat{Y},Y\right)=1-\frac{1}{2}\left(\text{SSIM}\left(\hat{Y},Y\right)+\text{SSIM}\left({\hat{Y}}_{↓},Y_{↓}\right)\right)$$

As shown in Equation 21, The Total Loss is the weighted sum of the three components:

(21) $$ℒ=\alpha {ℒ}_{int}+\beta {ℒ}_{grad}+\gamma {ℒ}_{ssim}$$

where *α*, *β*, and *γ* are the weighting coefficients for each component, with specific values detailed in the experimental section. The three loss functions provide complementary constraints: ${ℒ}_{int}$ penalizes pixel-level deviations, ${ℒ}_{grad}$ emphasizes local edge differences, and ${ℒ}_{ssim}$ encourages structural similarity between the predicted and target frames.

#### 2.2.7 Anomaly inference

During inference, the model takes *T* consecutive frames as input and predicts the next frame ${\hat{Y}}_{t}$. The deviation from the observed frame *Y_t_* is measured by the negative peak signal-to-noise ratio (PSNR). As shown in Equation 22, PSNR is computed from the pixel-wise mean squared error (MSE) via a logarithmic transformation, mapping reconstruction errors to the decibel scale. It effectively suppresses minor fluctuations in normal frames caused by stochastic texture variations, while being highly sensitive to large local errors that typically accompany anomalous motions or appearance changes. Consequently, the negative PSNR directly serves as a natural anomaly score, where larger prediction errors yield higher scores, following the common future-frame prediction setting in which larger prediction errors indicate stronger deviation from learned normal dynamics (Liu et al., 2018). Let MSE*_t_* denote the mean squared prediction error over the pixels and channels of frame *t*:

(22) $$S_{t}=-{\text{PSNR}}_{t}=-10\log_{10}\frac{L^{2}}{{\text{MSE}}_{t}},\;{\text{MSE}}_{t}=\frac{1}{N}{\hat{Y}}_{t}-Y_{t}2^{2},$$

where *L* is the dynamic range of the image representation and *N* is the number of pixel-channel values in a frame. Since the model is trained exclusively on normal videos, normal frames are expected to yield smaller prediction errors and lower anomaly scores, whereas anomalous frames tend to produce larger prediction errors and higher anomaly scores.

As shown in Equation 23, The anomaly scores are further smoothed using a Gaussian filter and normalized to [0,1] by Min–Max normalization:

(23) $${\bar{S}}_{t}=\frac{{\tilde{S}}_{t}-\underset{j}{\min}{\tilde{S}}_{j}}{\underset{j}{\max}{\tilde{S}}_{j}-\underset{j}{\min}{\tilde{S}}_{j}},$$

where ${\tilde{S}}_{t}$ and ${\bar{S}}_{t}$ denote the smoothed and normalized anomaly scores, respectively.

## 3 Experiments

### 3.1 Experimental settings

#### 3.1.1 Implementation details and hyperparameter configuration

The proposed TFMAD model is implemented in PyTorch. Given six consecutive RGB frames, the model predicts the immediately subsequent frame. Both the input frames and the target frame are resized to 256×256 pixels and normalized to the range [−1,1]. The model is optimized using AdamW with an initial learning rate of 5 × 10^−5^, which is progressively reduced according to a cosine annealing schedule. The hardware and software environment used in the experiments is summarized in **Table 3**.

**Table 3:** Hardware and software environment used in the experiments.

Item	Specification
Computing platform	AutoDL cloud computing container
Operating system	Ubuntu 20.04
GPU	NVIDIA GeForce RTX 4090
Python	3.8.20
PyTorch	2.1.2
CUDA	11.8
cuDNN	8.7.0

The main training and model hyperparameters are summarized in **Table 4**. Unless otherwise specified, all TFMAD variants in the ablation studies were trained using the same configuration to ensure a fair comparison.

**Table 4:** Training and model hyperparameter configuration.

Hyperparameter	Value
Input sequence length	6 frames
Prediction horizon	1 frame
Input resolution	256 × 256
Input normalization range	[−1,1]
Batch size	8
Training epochs	100
Optimizer	AdamW
Initial learning rate	5 × 10^−5^
Learning-rate schedule	Cosine annealing
Loss weights (*α,β,γ*)	(1.0,1.0,1.0)
PSTM scanning paths *K*	12
Block side length *P*	4
SSM state dimension *d*_state_	16

#### 3.1.2 Evaluation metrics and thresholding strategy

As described in the anomaly inference stage, the raw frame-level anomaly scores are smoothed and normalized to obtain ${\bar{S}}_{t}\in \left[0,1\right]$. As shown in Equation 24, Given a decision threshold *τ*, the continuous anomaly score is converted into a binary frame-level prediction as

(24) $${\hat{y}}_{t}=\{\begin{array}{ll} 1, & {\bar{S}}_{t}\geq \tau ,\\ 0, & {\bar{S}}_{t}<\tau , \end{array}$$

where ${\hat{y}}_{t}=1$ and ${\hat{y}}_{t}=0$ denote anomalous and normal frames, respectively.

1895982Based on the predicted and ground-truth labels, *TP*, *TN*, *FP*, and *FN* denote the numbers of correctly detected anomalous frames, correctly detected normal frames, normal frames incorrectly classified as anomalous, and missed anomalous frames, respectively. As shown in Equation 25, Accuracy, false-positive rate (FPR), and falsenegative rate (FNR) are defined as

(25) $$\text{Accuracy }=\frac{TP+TN}{TP+TN+FP+FN},\text{ FPR }=\frac{FP}{FP+TN},\text{ FNR }=\frac{FN}{FN+TP}.$$

Since these metrics depend on a predefined decision threshold, the frame-level Area Under the Receiver Operating Characteristic Curve (AUC) is adopted as the primary threshold-independent metric.

Both Micro-AUC and Macro-AUC are reported. Micro-AUC is calculated by concatenating the anomaly scores and ground-truth labels of all test videos before computing a single AUC, reflecting the overall discrimination performance. Macro-AUC is obtained by averaging the AUC values calculated independently for each test video, reflecting performance consistency across different scenarios.

To determine the operating threshold used in the ablation studies, a leave-one-video-out calibration was performed using the normalized anomaly scores of the complete TFMAD model. In each fold, five videos were used to select the threshold that maximized the difference between the true-positive rate and false-positive rate, while the remaining video was held out. The results are reported in **Table 5**. The calibrated thresholds exhibit only minor variation across the six folds. Accordingly, a fixed threshold of *τ* = 0.30 is applied to all ablation variants to ensure a consistent evaluation protocol.

**Table 5:** Leave-one-video-out threshold calibration results.

Held-out video	Threshold
Bird	0.2892
Blight	0.2892
Fire	0.3010
Lodging	0.2880
Vehicle	0.2892
Yellowing	0.2892
**Mean ± Std.**	**0.2910 ± 0.0045**

The bold value indicates the mean +/- standard deviation of the calibrated thresholds across the six held-out videos.

Micro-AUC and Macro-AUC are reported in both the comparative and ablation experiments, whereas Accuracy, FPR, and FNR are reported only in the ablation studies. Unless otherwise stated, all detection metrics are reported as percentages (%). Threshold-dependent metrics are not used for cross-method ranking because operating thresholds for external baselines are not calibrated in a strictly comparable way; therefore, threshold-independent AUC metrics are used as the primary basis for comparative evaluation.

The number of model parameters is reported in millions (M), while computational complexity is reported in giga floating-point operations (GFLOPs) per input sequence. FLOPs were calculated using fvcore with an input shape of 1 × *T* × 3 × 256 × 256, where *T* denotes the input sequence length. When the input sequence length was treated as an experimental variable, FLOPs were recalculated using the corresponding value of *T*. Inference speed is reported in frames per second (FPS), defined as the number of future frames predicted per second. FPS was measured under FP32 precision with a batch size of 1. For each configuration, 30 warm-up iterations were followed by 100 timed forward passes, with CUDA synchronization performed before and after timing. All models were profiled under identical hardware and software environments.

### 3.2 Ablation studies

Two ablation experiments were conducted to evaluate the functional modules and the number of scanning paths in PSTM.

#### 3.2.1 Ablation of functional modules

**Figure 7:**
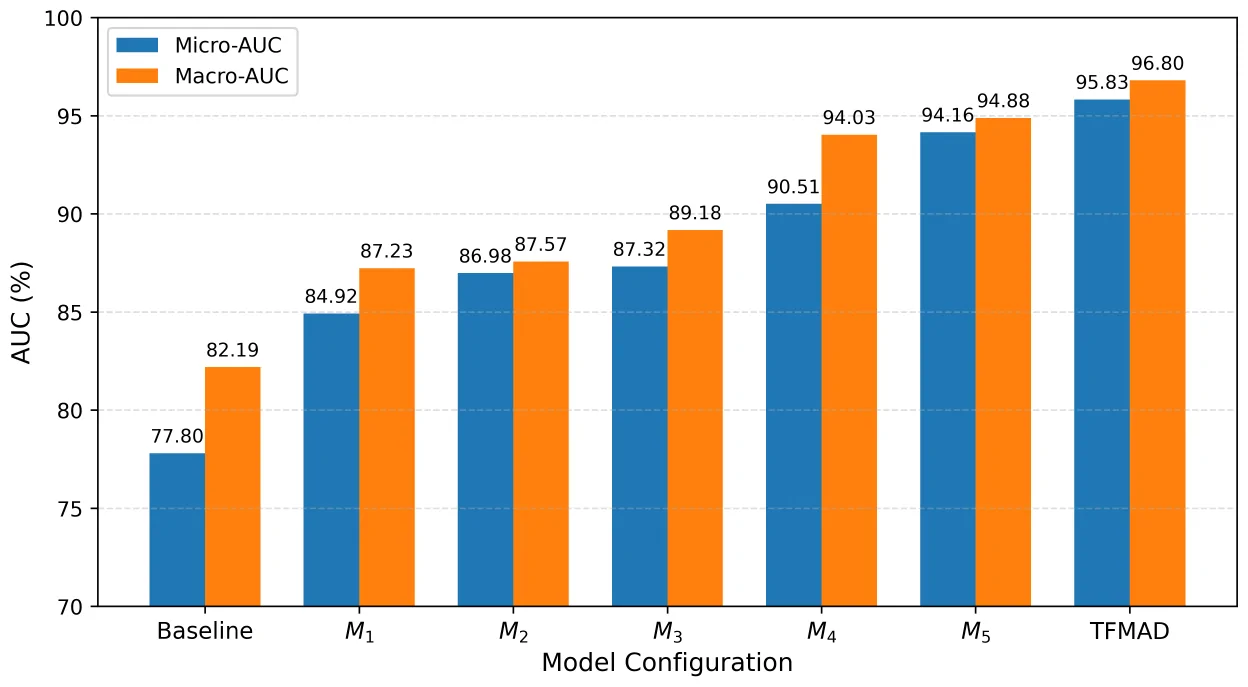
Micro-AUC and macro-AUC results of the module ablation study.

The contributions of TSSA, FSCM, PSTM, and DGF were evaluated using the configurations listed in **Table 6**. The corresponding Micro-AUC and Macro-AUC results are illustrated in **Figure 7**.

**Table 6:** Ablation results of the main functional modules.

Model	TSSA	FSCM	PSTM	DGF	Micro-AUC	Macro-AUC	Accuracy	FNR	FPR
Baseline	–	–	–	–	77.80	82.19	80.28	43.57	14.53
*M*_1_	✓	–	–	–	84.92	87.23	82.43	32.99	14.22
*M*_2_	✓	✓	–	–	86.98	87.57	84.63	23.63	13.58
*M*_3_	✓	✓	–	✓	87.32	89.18	85.62	20.59	13.03
*M*_4_	✓	–	✓	–	90.51	92.03	85.70	25.21	11.93
*M*_5_	✓	✓	✓	–	94.16	94.88	91.67	18.77	6.06
TFMAD	✓	✓	✓	✓	95.83	96.80	93.59	8.42	5.97

All metrics are reported in percentages.

The baseline achieves a Micro-AUC of 77.80% and a Macro-AUC of 82.19%. Introducing TSSA improves these values to 84.92% and 87.23%, respectively, while reducing the FPR from 43.57% to 32.99%. Adding FSCM further improves Micro-AUC to 86.98%, and the *M*_3_ configuration with DGF reaches a Macro-AUC of 89.18% and an FPR of 20.59%. The PSTM-based variant *M*_4_ achieves higher Micro-AUC and MacroAUC values of 90.51% and 92.03% and reduces FNR to 11.93%, but its FPR remains higher than those of *M*_2_ and *M*_3_. This indicates a trade-off between improved anomaly sensitivity and false-positive control under this configuration. Combining FSCM and PSTM in *M*_5_ increases Micro-AUC and Macro-AUC to 94.16% and 94.88%, respectively. The complete TFMAD model further achieves the best overall ablation results, with a Micro-AUC of 95.83%, a Macro-AUC of 96.80%, and an Accuracy of 93.59%. Compared with *M*_5_, adding DGF reduces the FPR from 18.77% to 8.42%, while the FNR changes slightly from 6.06% to 5.97%. Overall, TFMAD improves Micro-AUC and Macro-AUC over the baseline by 18.03 and 14.61 percentage points, respectively, and the ablation results suggest that the modules contribute most favourably when used together.

#### 3.2.2 Ablation of PSTM scanning paths

**Figure 8:**
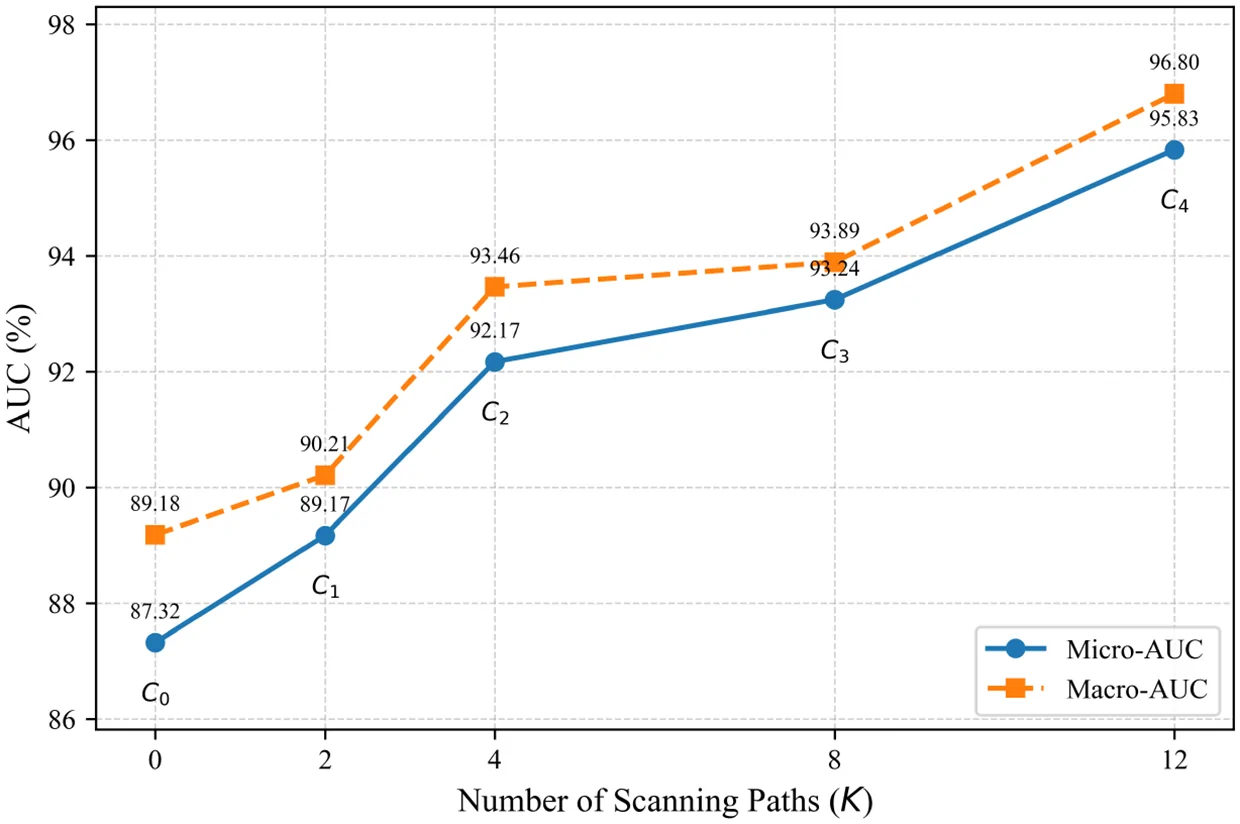
Micro-AUC and macro-AUC under different numbers of PSTM scanning paths *K*.

To evaluate the multi-path scanning strategy, the remaining components were fixed while varying the scanning configuration in PSTM. As shown in **Table 7** and **Figure 8**, *C*_0_ removes state-space scanning, whereas *C*_1_–*C*_4_ progressively introduce pixel-level scanning, reverse scanning, and block-level scanning.

**Table 7:** Ablation results of different scanning configurations in PSTM.

Configuration	Paths (*K*)	Scanning mode	Params	FLOPs	FPS	Micro-AUC	Macro-AUC
*C*_0_	0	None	21.25	19.66	58.77	87.32	89.18
*C*_1_	2	*S*1*,S*2	24.16	21.72	51.20	89.17	90.21
*C*_2_	4	*S*_1_*,S*_2_ w/Reverse	24.36	22.51	50.99	92.17	93.46
*C*_3_	8	*S*_1_–*S*_4_ w/Reverse	24.76	24.10	49.83	93.24	93.89
*C*_4_	12	*S*_1_–*S*_6_ w/Reverse	25.15	25.68	49.88	95.83	96.80

Introducing the two pixel-level temporal scanning paths in *C*_1_ improves Micro-AUC and Macro-AUC from 87.32% and 89.18% to 89.17% and 90.21%, respectively. Adding their reverse counterparts in *C*_2_ further raises the two metrics to 92.17% and 93.46%, suggesting that the reverse paths provide complementary information under the evaluated setting. The block-level scanning paths introduced in *C*_3_ and *C*_4_ are associated with further increases in both AUC metrics. The complete 12-path configuration achieves the best Micro-AUC and Macro-AUC of 95.83% and 96.80%, outperforming the 8-path configuration by 2.59 and 2.91 percentage points, respectively. Meanwhile, the parameter count increases from 24.76M to 25.15M, and the model maintains an inference speed of 49.88 FPS. Therefore, the 12-path configuration is adopted because it provides broader scanning coverage with limited additional computational cost in the reported experiments.

### 3.3 Parameter sensitivity analysis

We further evaluated the sensitivity of TFMAD to the input sequence length *T*, PSTM block size *P*, and SSM state dimension *d*_state_. In each experiment, only the parameter under investigation was varied, while all other settings were kept unchanged.

As shown in **Table 8**, *T* = 6 achieves the highest Micro-AUC, Macro-AUC, and Accuracy, together with the lowest FPR and FNR. Although *T* = 4 provides a higher inference speed, its detection performance is lower, while increasing the input length to eight frames reduces both performance and efficiency. Therefore, *T* = 6 is adopted as the default input length.

**Table 8:** Sensitivity to the input sequence length *T*.

Input length	FPS	Micro-AUC	Macro-AUC	Accuracy	FPR	FNR
4	53.67	93.14	96.21	92.86	11.44	6.21
6	49.88	95.83	96.80	93.59	8.42	5.97
8	40.94	91.75	94.86	87.85	13.69	11.82

**Table 9** shows that different block sizes favor different anomaly types. The smaller block size *P* = 2 performs well on localized dynamic anomalies such as Fire and Bird, whereas *P* = 8 is more effective for spatially extended anomalies such as Lodging and Blight. However, *P* = 4 achieves the highest overall Micro-AUC and Macro-AUC, indicating a better balance between fine-grained details and regional spatial context.

**Table 9:** Sensitivity to the PSTM block size *P*.

Block size *P*	Vehicle	Fire	Yellowing	Bird	Lodging	Blight	Micro-AUC	Macro-AUC
2	98.84	94.97	95.30	100.00	89.03	95.93	93.39	95.67
4	99.98	94.90	97.46	99.71	91.17	97.61	95.83	96.80
8	96.77	91.57	96.85	88.10	93.25	98.04	93.46	94.09

As reported in **Table 10**, reducing *d*_state_ to 8 slightly improves efficiency but noticeably decreases detection performance. Increasing it to 32 introduces more parameters and computation without further improving the AUC results. Therefore, *d*_state_ = 16 is selected as the default setting because it provides the best overall balance between detection performance and computational efficiency.

**Table 10:** Sensitivity to the SSM state dimension *d*_state_.

*d*_state_	Params (M)	GFLOPs	FPS	Micro-AUC	Macro-AUC
8	24.85	24.76	50.11	93.32	95.59
16	25.15	25.68	49.88	95.83	96.80
32	25.74	26.93	49.23	95.61	96.72

### 3.4 Comparative experiments

To comprehensively evaluate TFMAD, it is compared with seven representative video anomaly detection methods, including four general VAD approaches and three methods developed for UAV aerial scenarios. To the best of our knowledge, no publicly available method directly matches the complete task setting considered in this study, which combines normal-only learning, moving-UAV paddy-field videos, open category macroscopic anomalies, and frame-level evaluation. Therefore, the comparison focuses on representative general and aerial video anomaly detection methods with publicly available or reproducible implementations. The compared methods and their main characteristics are summarized in **Table 11**.

**Table 11:** Representative video anomaly detection methods used for comparison.

Category	Method	Main characteristic
General VAD	ASTNet (Le and Kim, 2023) MPN (Lv et al., 2021) VADMamba (Lyu et al., 2025)	Multi-scale spatial–temporal feature fusion Meta-prototype convolutional autoencoder Vector-quantized Mamba-based U-Net
	LGN-Net (Zhao et al., 2023)	Dual-branch spatial–temporal modeling
UAV-based VAD	ANDT (Jin et al., 2022) HSTforU (Le et al., 2025) ASTT (Tran et al., 2024)	Transformer-based video reconstruction Multi-scale spatial–temporal feature reconstruction Spatial–temporal feature disentanglement
	TFMAD (Ours)	Frequency disentanglement and state-space temporal modeling

#### 3.4.1 Implementation of the baseline methods

All baseline methods were retrained on URVAD using the official source code released by their authors. Only the data-loading and annotation interfaces were adapted to the URVAD directory structure, while the main model designs were retained. All methods used the same normal-only training split and test split. The RGB frames were resized to 256 × 256 pixels and normalized to [−1,1], and input sequences were generated within individual videos without crossing video boundaries.

For a consistent training budget, all methods were trained for 100 epochs with a batch size of 8. AdamW was used with cosine annealing and a weight decay of 2 × 10^−4^ for all methods. The temporal input length, backbone, loss configuration, and method-specific hyperparameters are summarized in **Table 12**. All results were recalculated using the same frame-level annotations and evaluation code for Micro-AUC and Macro-AUC.

**Table 12:** Implementation configurations of the compared methods on URVAD.

Method	Input	Initial LR	Method-specific settings
ASTNet	4→1	5 × 10^−5^	WiderResNet-38 (layer 6); *λ*_int_ = *λ*_grad_ = *λ*_SSIM_ = 1.
MPN	6→1	1 × 10^−4^	10 prototypes; feature and key dimensions of 128; *λ*_pix_ = 1 and *λ*_fea_ = *λ*_dis_ = 10−4.
VADMamba	6→1	2 × 10^−4^	Encoder and decoder depths of [1,1,1,1]; 50 VQ embeddings; *λ*MSE = *λ*_grad_ = *λ*VQ = 1.
ANDT	6→1	5 × 10^−5^	Patch size 16; embedding dimension 768; 12 Transformer layers; 12 attention heads; MLP dimension 3072; MSE loss.
HSTforU	4→1	2 × 10^−4^	$\lambda_{\mathrm{int}}=\lambda_{\text{grad}}=\lambda_{\text{MS-SSIM}}=\lambda_{L_{2}}=1.$
LGN-Net	4→1	5 × 10^−5^	Memory size 10; feature and key dimensions of 512; update and gathering temperatures of 0.1; *λ*_MSE_ = 1 and *λ*_comp_ = *λ*_sep_ = 0.01.
ASTT	6→1	5 × 10^−5^	Patch size 16; embedding dimension 768; 12 Transformer layers; 12 attention heads; MLP dimension 3072; dropout 0.1; *λ*_int_ = *λ*_grad_ = *λ*_SSIM_ = 1; gradient-clipping norm 1.0.

#### 3.4.2 Quantitative comparison

The frame-level detection results are reported in **Table 13**. TFMAD achieves the highest reported MicroAUC and Macro-AUC under the evaluated setting, with values of 95.83% and 96.80%, respectively. It also obtains the highest AUC values for Vehicle, Fire, and Bird anomalies, showing strong performance on categories that contain moving targets or rapidly changing abnormal events.

**Table 13:** Frame-level AUC comparison on the six anomaly categories.

Method	Vehicle	Fire	Yellowing	Bird	Lodging	Blight	Micro-AUC	Macro-AUC
ASTNet	77.98	90.86	98.15	94.96	87.73	94.40	89.45	90.68
MPN	93.53	73.64	96.67	89.81	88.45	98.93	88.29	90.17
VADMamba	98.07	89.26	91.41	99.60	85.82	91.07	86.67	92.53
LGN-Net	93.41	88.72	87.74	89.93	92.92	99.02	89.38	91.95
ANDT	69.34	91.87	93.79	76.17	87.54	70.02	78.22	81.45
HSTforU	58.36	91.81	99.12	97.83	87.45	98.49	84.28	88.84
ASTT	97.63	93.27	94.51	87.12	86.39	96.03	91.32	92.49
TFMAD (Ours)	99.98	94.90	97.46	99.71	91.17	97.61	95.83	96.80

All results are reported as percentages, and the best result in each column is highlighted in bold.

TFMAD does not achieve the best result in every individual category. For Yellowing, its AUC of 97.46% is slightly lower than that of HSTforU, while LGN-Net performs better on Lodging and Blight. These categories are dominated by relatively slow or static appearance changes. In particular, lodging usually appears as a gradual low-frequency structural deformation, which may be less emphasized by the highfrequency weighting in FSCM. Similarly, yellowing and blight mainly appear as color and local texture changes with limited temporal motion. These characteristics may partly explain why methods that focus more strongly on spatial appearance reconstruction obtain slightly better results for some of these categories. Nevertheless, TFMAD remains competitive in these categories and achieves the highest Micro-AUC and Macro-AUC, indicating a more balanced result across the reported metrics.

#### 3.4.3 Computational efficiency

**Table 14** compares detection performance and computational efficiency. TFMAD contains 25.15M parameters and requires 25.68G FLOPs per input sequence, while achieving an inference speed of 49.88 FPS. Although MPN and VADMamba are lighter or faster, their Micro-AUC values are lower than that of TFMAD. In contrast, methods such as ASTNet, ANDT, HSTforU, and ASTT require considerably more parameters or operate at lower inference speeds.

**Table 14:** Comparison of computational efficiency and detection performance.

Method	Params (M)	FLOPs (G)	FPS	Micro-AUC	Macro-AUC
ASTNet	243.53	1117.92	16.62	89.45	90.68
MPN	13.29	44.48	134.60	88.29	90.17
VADMamba	14.80	4.20	87.81	86.67	92.53
LGN-Net	22.75	204.68	23.74	89.38	91.95
ANDT	138.82	134.13	40.17	78.22	81.45
HSTforU	137.62	74.17	24.49	84.28	88.84
ASTT	223.76	36.97	23.21	91.32	92.49
TFMAD	25.15	25.68	49.88	95.83	96.80

Overall, TFMAD provides a favorable balance between detection accuracy, model complexity, and inference speed. The reported FPS indicates real-time processing capability under the experimental GPU environment, while deployment performance on resource-constrained UAV edge devices requires further evaluation.

### 3.5 Visualization analysis

To complement the quantitative evaluation, we further examine the detection behavior of TFMAD from spatial and frame-level perspectives. Prediction-error heatmaps are used to illustrate the spatial distribution of prediction discrepancies, while frame-level anomaly-score curves and ROC curves are used to characterize the temporal response and threshold-independent discrimination performance of the model.

#### 3.5.1 Spatial error distribution analysis

**Figure 9:**
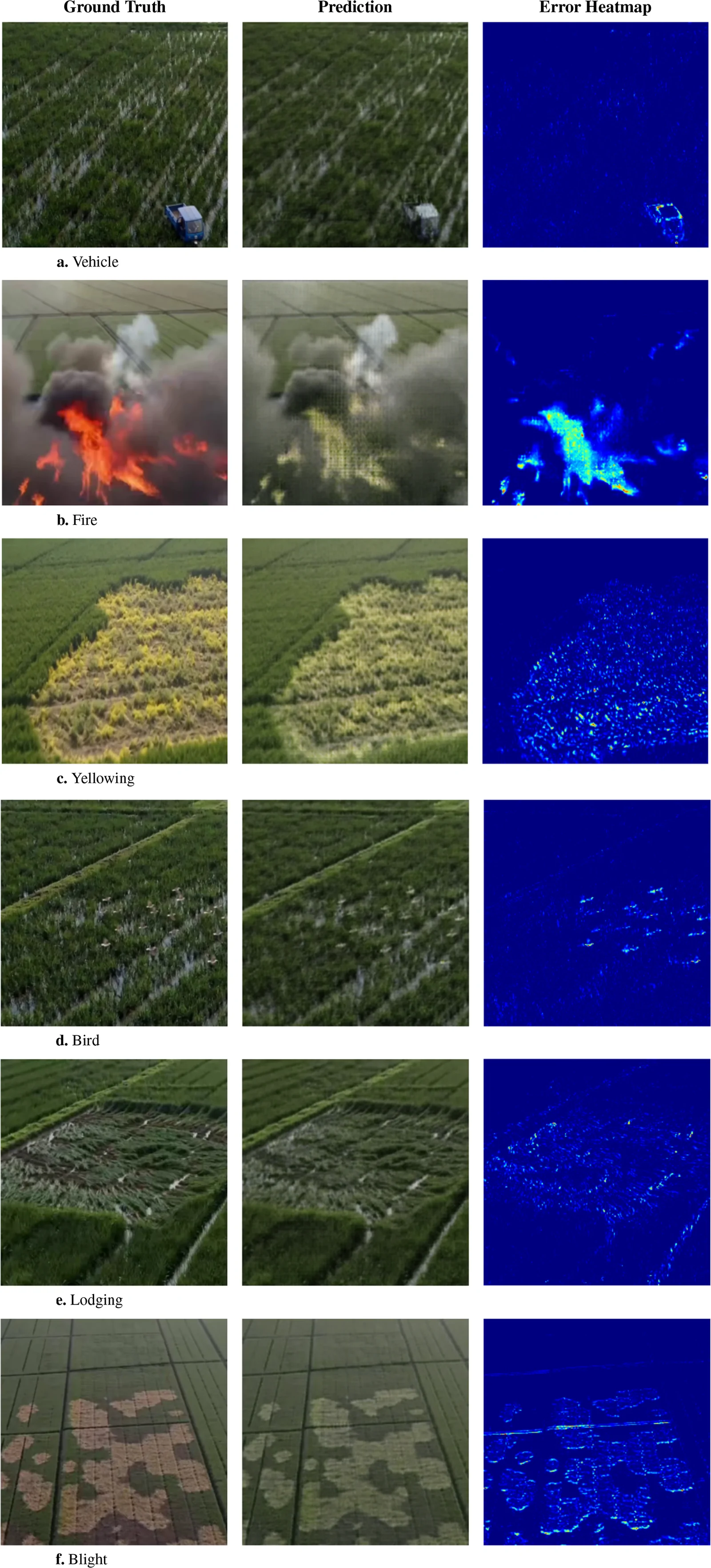
Qualitative visualization of the spatial prediction-error distributions under six typical anomaly scenarios. Each row presents the observed frame, predicted frame, and corresponding prediction-error heatmap from left to right. **(a)** Vehicle. **(b)** Fire. **(c)** Yellowing. The image continues on the next page. Qualitative visualization of the spatial prediction-error distributions. **(d)** Bird. **(e)** Lodging. **(f)** Blight.

**Figure 9** qualitatively compares the observed frames, predicted frames, and pixel-wise prediction-error heatmaps under six typical anomaly scenarios. In general, the model predicts the regular paddy-field background with relatively small errors, while producing stronger responses in regions containing unseen objects or abnormal appearance patterns.

For Vehicle and Fire, the error responses are mainly concentrated around the intruding vehicle and the dynamically changing flame and smoke regions, respectively. Yellowing and Blight produce more spatially diffuse responses because these anomalies are characterized by broad color and texture changes rather than compact foreground objects. Bird generates weaker and scattered responses owing to its small target size, whereas Lodging produces responses around the deformed crop structures and their boundaries. These observations indicate that TFMAD responds to both dynamic intrusions and appearance-based abnormalities, although small targets and gradual structural changes tend to produce less concentrated spatial error patterns.

It should be noted that the heatmaps provide only a qualitative visualization of the spatial distribution of prediction errors. Since the test set contains frame-level rather than pixel-level annotations, they are not used as a quantitative measure of anomaly localization accuracy. Moreover, the spatial concentration of an error response does not directly determine frame-level detection performance. A spatially diffuse or relatively weak response may still provide strong discrimination when it is consistently aggregated into the frame-level anomaly score.

#### 3.5.2 Temporal anomaly score analysis

**Figure 10:**
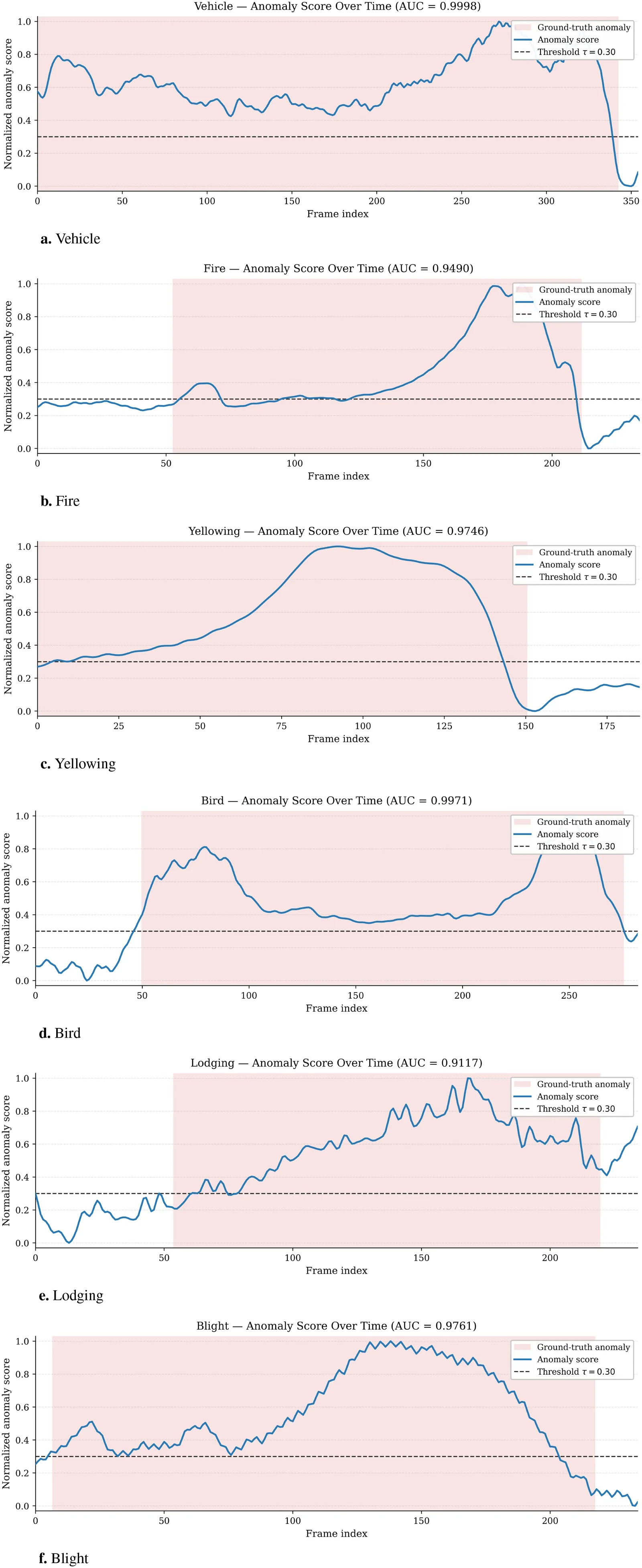
Frame-level normalized anomaly-score curves for six anomaly test videos. The blue curve represents the predicted anomaly score, the light-red shaded region indicates the ground-truth anomalous interval, and the dashed line denotes the operating threshold *τ* = 0.30. **(a)** Vehicle. **(b)** Fire. **(c)** Yellowing. The figure continues on the next page. Frame-level normalized anomaly-score curves. **(d)** Bird. **(e)** Lodging. **(f)** Blight.

**Figure 10** presents the normalized frame-level anomaly scores for the six test videos. The shaded regions indicate the ground-truth anomalous intervals, while the dashed line represents the fixed operating threshold *τ* = 0.30 used for supplementary binary evaluation.

For Vehicle, Bird, Yellowing, and Blight, the anomaly scores remain high during most anomalous intervals and decrease after the scenes return to normal, showing good temporal agreement with the annotations. In particular, although Bird produces relatively weak and scattered spatial errors, its frame-level scores remain clearly separated from those of normal frames. Similarly, the spatially diffuse responses of Yellowing and Blight are aggregated into discriminative frame-level anomaly scores.

In the Fire sequence, the score is relatively modest during the early stage of the anomalous interval and gradually increases as the flame and smoke become more pronounced, reaching its peak during the most severe stage of the event. The Lodging sequence exhibits greater score fluctuations and weaker separation near the anomaly boundaries. This may be attributed to its gradual structural deformation and relatively weak frame-to-frame changes, which can resemble normal variations in crop texture or UAV motion.

**Figure 11:**
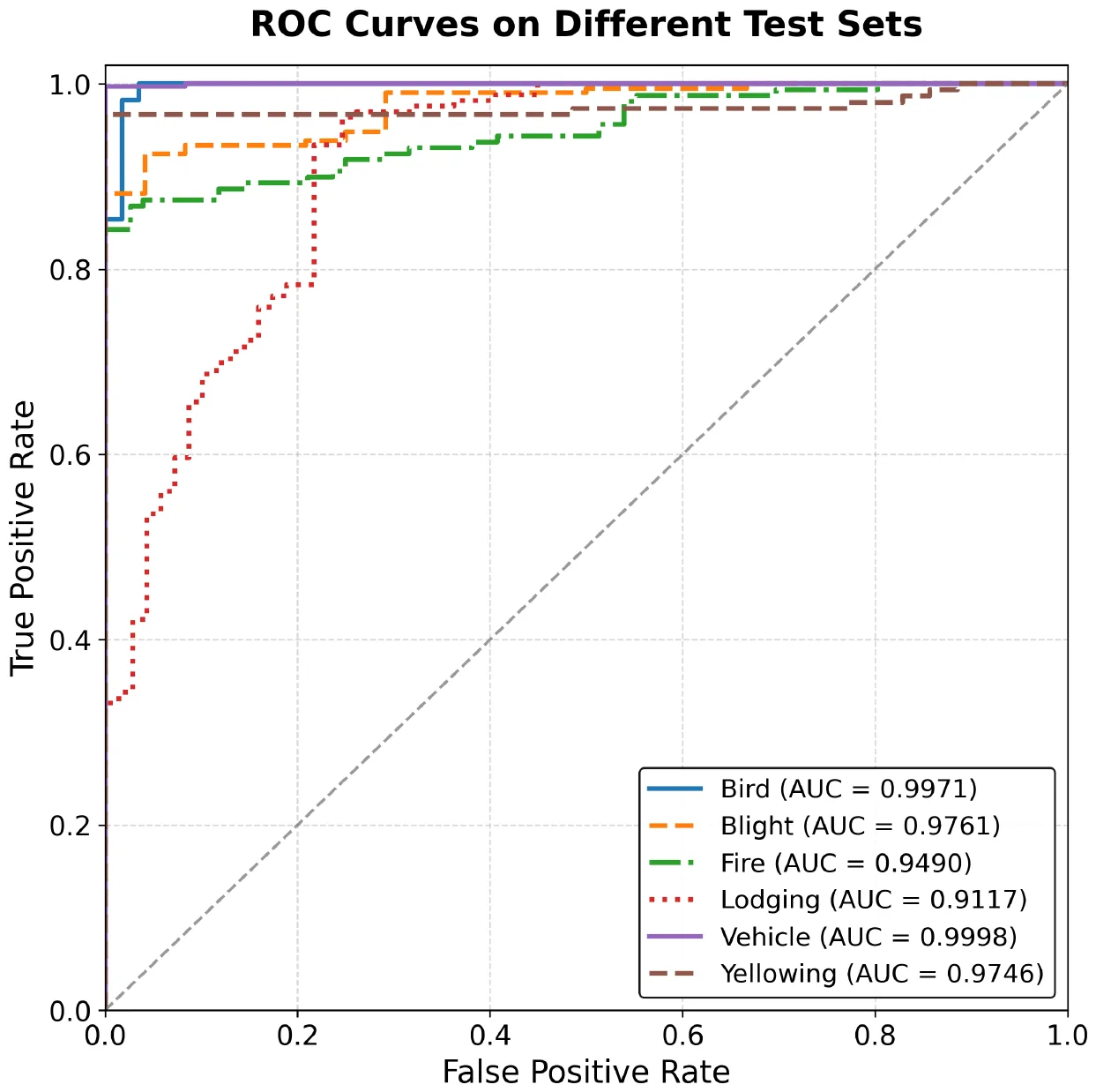
ROC curves on the different test sets, summarizing the overall discriminative performance of TFMAD across various anomaly scenarios.

The corresponding frame-level ROC curves are shown in **Figure 11**. Vehicle and Bird achieve nearperfect AUC values of 99.98% and 99.71%, respectively, while Yellowing and Blight also exhibit strong discrimination, with AUC values of 97.46% and 97.61%. Fire obtains an AUC of 94.90%, partly due to the weaker score separation during the early stage of the event. Lodging achieves the lowest AUC of 91.17%, consistent with its fluctuating scores and ambiguous anomaly boundaries. Overall, the temporal score curves and ROC results indicate that TFMAD distinguishes diverse anomaly types under the reported frame-level evaluation, although gradual structural anomalies remain comparatively challenging.

## 4 Discussion

The category-level results indicate that the effectiveness of TFMAD is related to the temporal characteristics of different anomalies. FSCM assigns relatively greater importance to temporal-frequency components with higher normalized frequencies and stronger spectral responses, making it particularly suitable for anomalies involving conspicuous motion or rapid appearance changes. For example, vehicles and birds generate localized motion patterns that differ substantially from regular crop dynamics, while flames and smoke exhibit rapidly changing shapes and textures. In contrast, lodging generally develops as a gradual and spatially extended deformation of the crop canopy. Its frame-to-frame variation is relatively weak and may overlap with normal canopy motion, viewpoint changes, or UAV ego-motion, which may partly explain why Lodging obtains the lowest category-level AUC of 91.17%. Nevertheless, this result does not demonstrate that FSCM completely suppresses low-frequency information, because the module retains a residual connection and operates in parallel with PSTM. The current observations therefore suggest a relative preference for prominent temporal variations rather than a direct causal relationship between frequency weighting and reduced lodging performance. Future work will investigate frequencyband ablation, learnable multi-band weighting, and explicit preservation of informative low-frequency components to improve sensitivity to slowly evolving structural anomalies.

The scale and distribution of URVAD limit the generalization of the current findings. The dataset contains seven normal training videos with 4,630 frames and six test videos with 1,565 frames, covering six representative macroscopic anomaly categories. Although these data support an initial evaluation of normal-only anomaly detection in UAV paddy-field videos, they do not systematically cover variations in weather, illumination, rice cultivar, growth stage, geographic region, flight altitude, or imaging platform. In addition, differences in anomaly duration result in unequal numbers of normal and anomalous frames across the test videos. Macro-AUC reduces the influence of videos containing larger numbers of frames, but it cannot fully remove the statistical uncertainty associated with the limited number of independent sequences. Future dataset development will therefore focus on collecting more independent events for each anomaly category, extending data acquisition across diverse agricultural and flight conditions, and reducing extreme video-level imbalance while preserving the natural rarity of abnormal events.

The reported computational efficiency should be interpreted according to the experimental hardware. TFMAD contains 25.15M parameters, requires 25.68G FLOPs per input sequence, and achieves 49.88 FPS using FP32 inference with a batch size of one on an NVIDIA GeForce RTX 4090. These results demonstrate real-time processing capability on the desktop GPU used in this study and indicate a favorable balance between detection performance and computational cost. However, they do not directly establish realtime operation on an onboard UAV platform, where memory capacity, power consumption, data-transfer latency, thermal constraints, and concurrent flight-control processes may affect end-to-end performance. The current model complexity indicates potential for edge-oriented optimization, but actual deployment performance must be verified on the target hardware. Future work will evaluate TFMAD on platforms such as NVIDIA Jetson and investigate mixed-precision inference, quantization, pruning, and hardware-aware model compression.

The current inference strategy also has methodological limitations. Negative PSNR provides a simple frame-level prediction-error score and is consistent with the future-frame prediction paradigm, but it is sensitive to global photometric variation and does not directly evaluate spatial localization quality. In addition, the ablation results support the usefulness of DGF within the proposed architecture, but they do not establish superiority over alternative skip-connection strategies such as skip pruning, explicit feature disentanglement, or adversarial constraints. These alternatives require separate controlled experiments and are therefore left for future work rather than being claimed as resolved in the present study.

## 5 Conclusion

This study presented TFMAD, a normal-only future-frame prediction framework for frame-level macroscopic anomaly detection in UAV paddy-field videos. By integrating frequency-aware spatiotemporal correlation modeling, multi-path state-space scanning, and discrepancy-guided feature fusion, TFMAD learns regular field dynamics from normal videos and identifies observations that deviate from the learned patterns. Experiments on URVAD show that the proposed method achieves 95.83% Micro-AUC and 96.80% Macro-AUC, obtaining the best overall performance among the compared methods under the reported experimental setting. These results demonstrate the feasibility of normal-only video anomaly detection for UAV-based paddy-field monitoring.

## Data Availability

The raw data supporting the conclusions of this article will be made available by the authors, without undue reservation.
